# Data from a survey on the impact of the pandemic on early-stage academics

**DOI:** 10.12688/openreseurope.13935.1

**Published:** 2021-11-17

**Authors:** Edyta Swider-Cios, Katalin Solymosi, Mangala Srinivas

**Affiliations:** 1 Young Academy of Europe (YAE), Munich, Germany; 2 Department of Cognitive Neuropsychology, Tilburg University, Tilburg, The Netherlands; 3 Department of Plant Anatomy, ELTE Eötvös Loránd University, Budapest, Hungary; 4 Hungarian Young Academy (HYA), Budapest, Hungary; 5 Department of Cell Biology and Immunology, Wageningen University and Research Centre, Wageningen, The Netherlands

**Keywords:** pandemic, researchers, survey, young academy

## Abstract

We would like to share data from a survey run by the Young Academy of Europe (YAE) from June to October 2020, with questions aiming to unravel the situation of early-career researchers (including early stage group leaders) working in Europe, during the COVID-19 pandemic. We were particularly interested in the impact of care activities (related to young children or other family members), and the impact of gender. We include the online survey and collected data, without identifying information. The survey is published in *Nature *Career Column (July, 2021) ( https://www.nature.com/articles/d41586-021-01952-6).

## Plain language summary

We are sharing data from an online survey run by the Young Academy of Europe (YAE) from June to October 2020, with questions aiming to understand the situation of early-career researchers during the COVID-19 pandemic.

## Methods

### The survey

A total of 33 questions were designed and uploaded onto the SoGoSurvey platform ( www.sogosurvey.com). The survey was quite detailed and contained three sections: working conditions (15 questions); personal background (13 questions); and optional questions (4 questions). The survey was a mix of multiple choice and short answer questions. It was tested with volunteers (board members of the YAE and members of the Hungarian Young Academy) for clarity before use. No changes were made after preliminary testing. As we are not experts in the field, we discussed the survey with experts during its development (see Acknowledgements).

A copy of the survey is available in the *Extended data* ^1^.

### Data collection

A link to the survey was shared via Young Academy of Europe (YAE) website ( https://yacadeuro.org/), newsletter, social media channels, and YAE contact channels (primarily other Young Academies, e.g. the Hungarian Young Academy and its Facebook page, where it was posted more than one time). We estimate our reach was between 1300-1500 people. We required that the respondent worked in research in academia in Europe at the time of the survey. Note that since we focused on Young Academies, this means that our participants were not completely random; most Young Academies tend to be quite selective in admission, but the selection criteria can vary by Academy. The survey results indicate whether or not a respondent was a member of a Young Academy.

At the end of the survey, participants were asked to provide informed consent. Information about the YAE Privacy Policy was included as well. The survey ran from June to October 2020.

Collected responses were then collated using Microsoft Excel (Microsoft Office Professional Plus 2019).

### Ethical considerations

Written informed consent for participation and publication of the participants’ details was obtained from the participants at the end of the online questionnaire. The specific text used was:

- I confirm that I am a responsible adult able to provide legal consent.
- I understand that my participation is voluntary, and I may withdraw at any time, without the need to provide any justification for it.
- I agree that my responses will be used as part of this study.
- I am aware that the data treatment will ensure my anonymity and I will not be identifiable.

Participants were asked to confirm consent at the end of the survey by ticking a ‘Certificate of Consent’ box.

No further ethical approval was sought, as this is deemed a low-risk study. Identifying information, such as names or institution names (where provided), have been removed from the shared data in order to maintain anonymity.

## Dataset presentation

We received a total of 151 responses. The entire data set, without identifying information, is presented as an Excel sheet and available in the *Underlying data*; responses to the open answer questions are also available in the *Underlying data* ^1^.

For more information about the dataset, please see our previous publication ^2^.

## Data availability

### Underlying data

Figshare: Young Academy of Europe (YAE) Survey on the Impact of COVID19 pandemic, https://doi.org/10.6084/m9.figshare.14971611.v1 ^1^.

This project contains the following underlying data:

- YAE COVID19 Survey Raw Data
- YAE COVID19 Survey Open Answers Results

### Extended data

Figshare: Young Academy of Europe (YAE) Survey on the Impact of COVID19 pandemic, https://doi.org/10.6084/m9.figshare.14971611.v1 ^1^.

This project contains the following extended data:

- YAE COVID19 Survey questionnaire

Data are available under the terms of the Creative Commons Attribution 4.0 International license (CC-BY 4.0).
