# Supplementary material for: Data from a survey on the impact of the pandemic on early-stage academics
Source: Open Res Eur. 2021 Nov 17;1:138. [Version 1] doi: 10.12688/openreseurope.13935.1 (PMC10446070; doi:10.12688/openreseurope.13935.1)
Supplement: Peer review history

# Peer review: Reviewer response for version 1

**DOI:** 10.21956/openreseurope.15019.r28482

**Published:** 2022-02-08

**Authors:** Tevfik Murat Yildirim

**Affiliations:** 1 University of Stavanger, Stavanger, Norway

This is a survey used by the authors to publish an article in the *Nature *career column. The dataset includes a large number of questions that are designed to advance our understanding of how the COVID-19 pandemic has affected early-career researchers in Europe. The sample size is large enough for statistical analysis, and the questions utilized in the dataset are sound. On the other hand, the low response rates reported by the authors, as well as the study's exclusive focus on 'young academies', necessitate the use of caution when interpreting the results from the study. Despite these relatively minor weaknesses, however, the dataset will surely contribute to our understanding of the pandemic and its consequences for academics.

Are sufficient details of methods and materials provided to allow replication by others?

Yes

Is the rationale for creating the dataset(s) clearly described?

Yes

Are the datasets clearly presented in a useable and accessible format?

Yes

Are the protocols appropriate and is the work technically sound?

Yes

Reviewer Expertise:

gender and politics

I confirm that I have read this submission and believe that I have an appropriate level of expertise to confirm that it is of an acceptable scientific standard.

# Response

**Published:** 2022-02-08

**Authors:** Mangala Srinivas

**Affiliations:** Wageningen University and Research Centre, The Netherlands

Thank you for your comments. We agree that the data needs to be interpreted carefully, and hope it will be useful.

# Peer review: Reviewer response for version 1

**DOI:** 10.21956/openreseurope.15019.r28057

**Published:** 2021-12-08

**Authors:** Karolina Lendak-Kabók

**Affiliations:** 1 University of Novi Sad, Novi Sad, Serbia

The data note title: Data survey on the impact of the pandemic on early-stage academics is a timely contribution which sheds light on the early career researchers (ECR) who are in a precarious situation in the neoliberal academic arena. ECRs are in a vulnerable position, they are in a period of life when they are starting a family, while they are expected to put a considerable amount of effort in their academic career, in order to stay on the demanding scientific track. Most of the ECRs (this is especially true for women) are struggling with finishing their PhDs while having small kids, or they are postponing motherhood in order to finish their PhDs. The pandemic put a lot of stress on ECRs, especially young academic mothers and the consequences of the pandemic on female ECRs are yet to be disclosed and analysed. Thus, the research presented through this data note is of great importance, as we need to raise awareness on ECRs and introduce various measures which will alleviate their career hurdles.

Are sufficient details of methods and materials provided to allow replication by others?

Yes

Is the rationale for creating the dataset(s) clearly described?

Yes

Are the datasets clearly presented in a useable and accessible format?

Yes

Are the protocols appropriate and is the work technically sound?

Yes

Reviewer Expertise:

Gender studies, women in academia, ECRs

I confirm that I have read this submission and believe that I have an appropriate level of expertise to confirm that it is of an acceptable scientific standard.

# Response

**Published:** 2021-12-08

**Authors:** Mangala Srinivas

**Affiliations:** Wageningen University and Research Centre, The Netherlands

Thank you for your comments! We hope these data will be useful for many researchers!
